# A two-dimensional mathematical model of non-linear dual-sorption of percutaneous drug absorption

**DOI:** 10.1186/1475-925X-4-40

**Published:** 2005-07-03

**Authors:** K George

**Affiliations:** 1 School of Information Systems, Computing and Mathematics; Department of mathematical Sciences, Brunel University, Uxbridge, Middlesex, , UB8 3PH, UK; 2 Aditi College, University of Delhi, Bawana, Delhi, 110039, India; 3 Civil and Computational Engineering Centre, School of Engineering, Singleton Park, Swansea, SA2 8PP Wales, UK

## Abstract

**Background:**

Certain drugs, for example scopolamine and timolol, show non-linear kinetic behavior during permeation process. This non-linear kinetic behavior is due to two mechanisms; the first mechanism being a simple dissolution producing mobile and freely diffusible molecules and the second being an adsorption process producing non-mobile molecules that do not participate in the diffusion process. When such a drug is applied on the skin surface, the concentration of the drug accumulated in the skin and the amount of the drug eliminated into the blood vessel depend on the value of a parameter, *C*, the donor concentration. The present paper studies the effect of the parameter value, *C*, when the region of the contact of the skin with drug, is a line segment on the skin surface. To confirm that dual-sorption process gives an explanation to non-linear kinetic behavior, the characteristic features that are used in one-dimensional models are (1) prolongation of half-life if the plot of flux *versus *time are straight lines soon after the vehicle removal, (2) the decrease in half-life with increase in donor concentration. This paper introduces another feature as a characteristic to confirm that dual-sorption model gives an explanation to the non-linear kinetic behavior of the drug. This new feature is "the prolongation of half-life is not a necessary feature if the plots of drug flux *versus *time is a non-linear curve, soon after the vehicle removal".

**Methods:**

From biological point of view, a drug absorption model is said to be nonlinear if the sorption isotherm is non-linear. When a model is non-linear the relationship between lag-time and donor concentration is non-linear and the lag time decreases with increase in donor concentration. A two-dimensional dual-sorption model is developed for percutaneous absorption of a drug, which shows non-linear kinetic behavior in the permeation process. This model may be used when the diffusion of the drug in the direction parallel to the skin surface must be examined, as well as in the direction into the skin, examined in one-dimensional models. The dual-sorption model is an initial/boundary value problem which consists of (1) one non-linear, two-dimensional, second-order parabolic equation, (2) boundary conditions, (3) one initial condition. Note that, the number of boundary conditions are, six and four, respectively, if the permeation process under consideration is, during the application of the vehicle and during the removal of the vehicle. Adopting the approach of method of lines, the initial/boundary value problem is transformed into an initial-value problem, which consists of (1) a system of non-linear ordinary differential equations, (2) one initial condition. The system of non-linear ordinary differential equations contains time-dependent non-homogeneous terms, if the permeation process under consideration is, during the application of the vehicle. To solve this initial-value problem, an eight-stage sequential algorithm which is second-order accurate, and requires only tri-diagonal solvers, is developed.

**Results:**

Simulation of the numerical methods described is carried out with various values of the parameter *C*. The illustrations are given in the form of figures. The concentration profiles are viewed as parabolas along the mesh lines parallel to *x*-axis or *y*-axis. The flow rates in different subregions of the skin-region are studied. The shapes of the concentration profiles are examined before and after the steady-state concentration is reached. The concentration reaches steady-state when the flux reaches the steady state. The plots of flux *versus *time and cumulative amount of drug eliminated into the receptor cell *versus *time are given.

**Conclusion:**

Based on the various values of the parameter, *C*, conclusions are drawn about (1) flow rate of the drug in different regions of the skin, (2) shape of the concentration profiles, (3) the time required to reach the steady-state value of the concentration, (4) concentration of the drug in different regions of the skin, when steady-state value of the concentration is reached, (5) the time required to reach the steady-state value of the flux, (6) time required to reach the steady-state value of the concentration of the drug, (7) half-life of the concentration of the drug and (8) lag-time.

A comparison, between this two-dimensional model and the one-dimensional non-linear dual-sorption model that exists in the literature, is done based on (1) the shape of the concentration profiles at various time levels, (2) the time required to reach the steady-state value of the concentration, (3) lag-time and (4) half-life.

## Background

A non-linear model takes into account the property of the skin to sorb and bind substances during the process of permeation in addition to the ordinary dissolution. Such a non-linear model can be used to explain the disparity between the steady-state diffusivity of the drug in the skin and the unsteady-state value computed from transient (time-lag) permeation experiments, see [1]. A non-linear dual-sorption model of percutaneous drug absorption is described in [2]. Another mathematical model of percutaneous drug absorption and its exact solution is described in [3]. In [4], the non-linear percutaneous permeation kinetics of timolol is studied *in vitro *with human cadaver skin. A model for a suspension with a finite dissolution rate is solved numerically in [5]. All these non-linear models are one-dimensional and the region of contact of the skin with the drug, is a single point, say *x *= 0, where *x *measures the distance into the skin.

To the authors' knowledge, no two-dimensional non-linear mathematical model of percutaneous drug absorption exists in the literature, where the region of contact is a line segment, say *x *= 0, 0 ≤ *y *≤ *L*_*c*_. The purpose of this paper is (1) to model the above situation mathematically (2) to obtain an efficient numerical method which can use a large time step compared to spacial discretization step; and also handles discontinuities between initial and boundary conditions in the mathematical model.

## Methods

### Model equations

#### (a) Concentration before the steady state is reached (Concentration during the application of the vehicle)

Let the drug be applied as an ointment on the skin-surface, say, {(0, *y*) : 0 ≤ *y *≤ *L*_*c*_}, at time *t *= 0. The two-dimensional model developed in [6] takes into account the drug kinetics in the skin where the ointment is not directly applied. The one-dimensional non-linear dual-sorption model given in [2] postulates the total concentration of the drug, *C*_*T*_, in the skin is composed of two parts, (1) the mobile solute concentration *C*_*D*_, which is due to the mechanism of simple dissolution and is expressed as *C*_*D *_= *K*_*D*_*C*, in which *K*_*D *_is skin/receptor cell partition coefficient and *C *is donor-cell concentration, (2) the immobile solute concentration *C*_*I*_, which is due to the adsorption process, and is expressed as , in which  is Langmuir's saturation constant and *b *is Langmuir's affinity constant. Hence the total concentration *C*_*T*_, in the skin is given by

*C*_*T *_= *C*_*D *_+ *C*_*I*_.

Based on [6] and [2], to describe the drug kinetics at any time *t > *0, a two-dimensional non-linear dual-sorption model is developed. Let the thickness (distance between the skin-surface and skin-capillary boundary) of the skin be *L*_*s*_. Assuming that skin is an isotropic medium, that is, the diffusivity *κ*, is the same in the *x *and *y *directions, the drug concentration *C*_*D *_= *C*_*D*_(*x*, *y*, *t*) in the skin is governed by



where *L*_*s*_, *L*_*d*_, *L*_*u *_and *L*_*c *_are positive real numbers. Note that, (1) is non-singular as *b *> 0,  > 0 and *C*_*D *_≥ 0. Following [2], the concentration along the skin-receptor cell boundary may be considered to have the value zero. Assuming that the donor-cell concentration at the uppermost *epidermis *{(0, *y*, *t*) : 0 ≤ *y *≤ *L*_*c*_, *t *> 0} is maintained at the value *C*, the boundary conditions for the model are given by



*C*_*D *_(0, *y*, *t*) = *K*_*D*_*C*, 0 ≤ *y *≤ *L*_*c*_, *t *> 0,     (2b)







*C*_*D *_(*L*_*s*_, *y*, *t*) = 0, -*L*_*d *_≤ *y *≤ *L*_*u*_, *t *> 0,     (2f)

Assuming that there is no drug in the skin before the application, the initial distribution associated with the PDE is

*C*_*D*_(*x*, *y*, 0) = 0,0 ≤ × ≤ *L*_*s*_, -*L*_*d *_≤ *y *≤ *L*_*u *_    (3)

The flux *J *= *J*(*t*), through the skin to the receptor site, per unit area, is given by



The cumulative amount of drug eliminated into the receptor cell per unit area at time *τ *(see [2]) is



The initial/boundary-value problem given by (1) to (3) is named as "problem (PA)".

#### (b) Concentration after the steady state is reached (Concentration after the vehicle removal)

Let *T*_*s *_denote the time at which the steady-state is reached by the problem (PA). Suppose that at time *t *= *T*_*s*_, the vehicle is removed instantaneously from the skin. Then (1) remains unchanged, but the initial conditions and boundary conditions changes and consequently, (2a) to (2c) disappear from the mathematical model, leaving the PDE's









*C*_*D *_(*L*_*s*_, *y*, *t*) = 0, -*L*_*d *_≤ *y *≤ *L*_*u*_, *t *> *T*_*s *_    (7d)

The initial distribution associated with PDE (6) is

*C*_*D *_= *C*_*D *_(*x*, *y*, *T*_*s*_), 0 ≤ *x *≤ *L*_*s*_, -*L*_*d *_≤ *y *≤ *L*_*u*_,     (8)

where *C*_*D *_(*x*, *y*, *T*_*s*_) is computed by solving problem (PA). The initial/ boundary-value problem given by (6) to (8) is named as "problem (PB)".

### Numerical methods

The approach to be adopted in obtaining a numerical solution, is the method of lines in which the initial/ boundary-value problem to be solved is transformed into a first-order initial-value problem. At any time *t > *0, the region associated with the skin is given by *R_s _*= {(*x*, *y*) : 0 ≤ *x *≤ *L*_*s*_, - *L*_*d *_≤ *y *≤ *L*_*u*_}. The region of the contact *R*_*c*_, of the drug and the skin surface is defined as *R*_*c *_= {(0, *y*) : 0 ≤ *y *≤ *L*_*c*_}. Superimpose on *R*_*s *_a rectangular grid with mesh lengths, *h *> 0 and *H *> 0, respectively, in the *x *and *y *directions. Let *k *> 0 be a constant time step. Let *N *be a positive integer, and *h *= 1/(*N *+ 1). Also it is assumed that *L*_*c *_= *Q*_*c*_*H*, *L*_*u *_= *Q*_*u*_*H*, *L*_*d *_= *Q*_*d*_*H*, where *Q*_*c*_, *Q*_*u *_and *Q*_*d *_are positive integers. The grid points are given by (*x*_*l*_, *y*_*m*_, *t*_*j*_); *x*_*l *_= *lh*, *l *= 0(1)(*N *+ 1), *y*_*m *_= *mH*, *m *= -*Q*_*d*_(1)*Q*_*u *_and *t_j_*= *jk*, *j *= 0, 1, 2, ...*s*, ...*etc*. with *sk *= *T*_*s*_. The values *t*_*j *_represent the time-levels for the problems (PA) and (PB) for *j *≤ *s *and *j *≥ *s *respectively. The finite-difference solution which approximates the solution *C*_*D *_(*x*, *y*, *t*) of the two-dimensional parabolic equation (1), is sought at each mesh point (*x*_*l*_, *y*_*m*_, *t*_*j*_) in the region [*R*_*s*_- *R*_*c *_- {(*L*_*s*_, *y*), -*L*_*d *_≤ *y *≤ *L*_*u*_}] × *t *> 0. Note that, for the initial-value problem (PB), *R*_*c *_= Φ (the empty set). For the problem (PA), the differential equation (1), subject to the boundary conditions (2a) to (2f), is discretized at all grid points in the region [*R*_*s *_- *R*_*c *_-{(*L*_*s*_, *y*), -*L*_*d *_≤ *y *≤ *L*_*u*_}] × [0 ≤ *t *≤ *T*_*s*_]. For the problem (PB), the differential equation (6), subject to the boundary conditions (7a) to (7d), is discretized at all grid points in the region [*R*_*s *_-{(*L*_*s*_, *y*), -*L*_*d *_≤ *y *≤ *L*_*u*_}] × [*t *> *T*_*s*_]. For notational simplicity, denote



#### Discretization for problem (PA)

At any time-level *t *= *t*_*j*_,  or , the {(*Q*_*d *_+ *Q*_*u *_+ 1) (*N *+ 1) - (*Q*_*c *_+ 1)} elements will be ordered in rows parallel to the *x*-axis and in vector form, and will be denoted by ***C***_***D***_(*t*) and ***Q***(*t*), where ***Q***(*t*) is due to the boundary condition (2b). For ***W ***= ***C***_***D ***_and ***Q***, and  =  or , denote



In the above notation *n *takes the value 0 if *m *≠ 0(1)*Q*_*c *_and takes the value 1 otherwise. The vector ***C***_***D***_(*t*) is a vector of unknowns and the vector ***Q***(*t*) is given by



All other  are zero. In the following discretizations, (*x*_*l*_, *y*_*m*_), *p*_*l*,*m*_, *q*_*l*,*m *_and *C*_*Dl*,*m*_, respectively, denote (*x*_*l*_, *y*_*m*_, *t*_*j*_), ,  and . At any time-level *t *= *t*_*j*_, the differential equation (1) can be discretized as



If  and  are respectively, suitable approximations to *C*_*Dxx*;*l*;*m *_and *C*_*Dyy*;*l*;*m*_, the above discretization can be written as



At an interior grid point (*x*_*l*_*, y*_*m*_),  and  are given by



If (*x*_*l*_, *y*_*m*_) is a boundary point, using the boundary conditions (2a) to (2f),  and  are given by



where

*A*_1 _= 0, *B*_1 _= 2, if *l *= 0, *m *= -*Q*_*d*_(1)(-1), (*Q*_*c *_+ 1)(1)*Q*_*u*_,

*A*_1 _= 0, *B*_1 _= 1, if *l *= 1, *m *= 0(1)*Q*_*c*_,

*A*_1 _= 1, *B*_1 _= 1, if *l *= 1(1)*N *- 1, *m *= -*Q*_*d*_(1)*Q*_*u*_,

*A*_1 _= 1, *B*_1 _= 0, if *l *= *N*, *m *= -*Q*_*d*_(1)*Q*_*u*_,

*A*_2 _= 0, *B*_2 _= 2, if *l *= 0(1)*N*, *m *= -*Q*_*d*_,

*A*_2 _= 2, *B*_2 _= 0, if *l *= 0(1)*N*, *m *= *Q*_*u*_,

*A*_2 _= 1, *B*_2 _= 1, if *l *= 0, *m *= (-*Q*_*d *_+ 1)(1)(-2), (*Q*_*c *_+ 2)(1)(*Q*_*u *_- 1),

*A*_2 _= 1, *B*_2 _= 0, if *l *= 0, *m *= -1

*A*_2 _= 0, *B*_2 _= 1, if *l *= 0, *m = *(*Q*_*c *_+ 1).

#### System of ordinary differential equations

Combining the discretizations (9) together with expressions for  and  given by (10) and (11) respectively, a system of ordinary differential equations is formed as



where



The value of *m *in *E*_**d **_and *F*_**d **_is -*Q*_*d*_, and that in *E*_**u **_and *F*_**u **_is *Q*_*c *_+ 1. The abbreviations *m*1, *m*2, ... etc. represent *m *+ 1, *m *+ 2, ... etc. For *m *≠ 0, 1, 2, ...*Q*_*c*_, *E*_*m *_and *F*_*m *_are square matrices of order *N *+ 1 and are given by



For *m *= 0, 1, 2, ..., *Q*_*c*_, *E*_*m *_and *F*_*m *_are square matrices of order *N *and are given by



For the values of *m *= -1, *Q*_*c *_+ 1, and *m *= 0, *Q*_*c*_, the matrices *G*_*m *_are of orders (*N *+ 1) × *N *and *N *× (*N *+ 1), respectively, and are given by



For the values of *m *= 0, 1, ..., *Q*_*c*_, *m *≠ 0, 1, 2, ...*Q*_*c*_, the matrices *O*_*m *_are the zero matrices of orders *N *and *N *+ 1 respectively. The matrices *O*_*d *_and *O*_*c *_are zero matrices of orders (*N *+ 1) × *N *and *N × *(*N *+ 1), respectively.

#### Recurrence relation and its implementation via sequential algorithm

In [7], an eight stage sequential algorithm is described to solve two space linear parabolic equations. In [2], a sequential algorithm of two tridiagonal solvers is described to solve one-space non-linear dual-sorption model. To the author's knowledge, there is no sequential algorithm in the literature to solve the two-dimensional non-linear parabolic equation (1). The aim of this section is to develop an eight-stage sequential algorithm of tridiagonal solvers, to solve the system of non-linear ordinary differential equations given by (12), which gives the solution of two-space non-linear parabolic equation (1). The main idea used is, to rewrite the system of equations (12) in such a way that, the numerical techniques described in [7] and [2] can be extended to two-space non-linear equations. To achieve this, we proceed as follows.

Let ***Q***(*t*) = ***R***(*t*) + ***S***(*t*), where the elements of the vectors ***R***(*t*) and ***S***(*t*) are, respectively, denoted by  and , and are defined by



Note that the vector, ***R***(*t*), is the contribution of *C*_*Dxx *_to the vector ***Q***(*t*). The vector, ***S***(*t*), is the contribution of *C*_*Dyy *_to the vector ***Q***(*t*). The system of non-linear ordinary differential equations given by (12) can be written as



It is known (see [2,8]) that the system of ordinary differential equations given by (12), subject to the initial condition (3), satisfies the recurrence relation



where D* = diag{d/d*t*}. The exponential term in the recurrence relation (16) will be approximated by its (2, 0) Padé approximant to give



which, following pre-multiplication, gives



Using the split form (15), and following [7] and [2], a four-stage sequential algorithm is developed to obtain , which is an approximation to ***C***_***D***_(*t *+ *k*) in (17). It is given by

(I - *r*_1_*kE*) ***Z ***= ***C***_***D***_(*t*) + *r*_1_*k****R***(*t*),     (18a)

(I - *r*_2_*kE*) ***V ***= ***Z ***+ *r*_2_*k****R***(*t*),     (18b)

(I - *r*_1_*kF*) ***Z ***= ***V ***+ *r*_1_*k****S***(*t*),     (18c)

(I - *r*_2_*kF*)  (t + k) = ***Z + ****r*_2_*k****S ***(*t*).     (18d)

where  (see [8])

Another solution, , which is an approximation to ***C***_***D***_(*t *+ *k*) in (17), is obtained by interchanging the matrices *E *and *F*; and the vectors ***R ***and ***S***; in (18a) to (18d). It is given by

(I - *r*_1_*kF*) ***Z ***= ***C***_*D*_(*t*) + *r*_1_*k****S***(*t*),     (18e)

(I - *r*_2_*kF*) ***V ***= ***Z ***+ *r*_2_*k****S***(*t*),     (18f)

(I - *r*_1_*kE*) ***Z ***= ***V ***+ *r*_1_*k****R***(*t*),     (18g)

(I - *r*_2_*kE*)  (t + k) = ***Z****+ r*_2_*k****R ***(*t*).     (18h)

Note that the approximations,  and , given by (18d) and (18h), are first-order accurate in time and their linear combination defined by



is second order accurate in time. The eight-stage algorithm, defined by (18a) to (18i) is, *L*_0 _stable and uses only tridiagonal solver.

#### Initial/boundary value problem (PB)

The "problem (PB)" is modelled by the equations (6) to (8). At any time-level t = t_*j*_, *j *≥ *s*, , the (*Q*_*d *_+ *Q*_*u *_+ 1) × (*N *+ 1) elements, will be ordered in rows parallel to the *x*-axis and in vector form, and will be denoted as ***C***_***D***_(*t*), with



At a grid point *x*_*l*_, *y*_*m*_, the required discretization is



For *l *≠ 0, *m *≠ (-*Q*_*d *_+ 1) (1) (*Q*_*u *_- 1);  and  are given by (10) and for *l *= 0, *m *= (-*Q*_*d *_+ 1)(1)(*Q*_*u *_- 1),

 and 

Combining the discretizations given by (19), a system of differential equations is formed as



in which



In the above matrices, the value of *m *is -*Q*_*d *_and *m*1 = *m *+ 1, *m*2 = *m *+ 2,... The matrices *E*_*i *_and *F*_*i*_, *i *= -*Q*_*d*_(1)*Q*_*u*_, are square matrices of order *N *+1, and are given by (13). Proceeding as in problem (PA), the eight-stage algorithm to solve the system of differential equations (20), subject to the initial conditions (8), is

(*I *- *r*_1_*kE*) ***Z ***= ***C***_***D***_(*t*),     (21a)

(*I *- *r*_2_*kE*) ***V ***= ***Z ***,    (21b)

(*I *- *r*_1_*kF*) ***Z ***= ***V***,     (21c)

(*I *- *r*_2_*kF*)  (t + k) = ***Z***,     (21d)

(*I *- *r*_1_*kF*) ***Z ***= ***C***_***D***_(*t*),     (21e)

(*I *- *r*_2_*kF*) ***V ***= ***Z***,     (21f)

(*I *- *r*_1_*kE*) ***Z ***= ***V***,     (21g)

(*I *- *r*_2_*kE*)  (t + k) = ***Z***,     (21h)



Note that the sequential algorithm defined by (21a) to (21i) is obtained from the equations (18a) to (18i), replacing the vectors ***R***(*t*) and ***S***(*t*), by the zero vector of length (*Q*_*d *_+ *Q*_*u*_+ 1) × (*N *+ 1). This shows the efficiency of the splitting of the vector ***Q***(*t*), and the eight-stage algorithm developed in problem (PA).

### Stability Analysis

The stability analysis of two-stage sequential algorithm for the solution of one-space non-linear second-order parabolic equation is described in [2]. The stability analysis of four-stage sequential algorithm for the solution of two-space second-order parabolic equation is described in [7]. In this section, following [7] and [2], it is to prove that the eight-stage sequential algorithms described by (18a) to (18i) and (21a) to (21i) are *L*_0 _stable.

The amplification matrix R*(*k*(*E *+ *F*)) of the method defined by (18a) to (18d) is given by



neglecting the higher order terms *O *(-*k*^3^).

Hence the symbol R*(-*z*), of the method defined by (18a) to (18d), to evaluate  (t + k) is given by



Similarly the symbol *R*^+^(-*-z*), of the method defined by (18e) to (18h), to evaluate (*t *+ *k*) is given by



In (22) and (23), *z *= -*kλ *where *λ *is an eigen value of the matrix *E *+ *F*. Hence using (22) and (23) the symbol, *S*(-*z*), of the method (18i) is



The method is *L*_0 _stable if

| *S*(-*z*) | ≤ 1     (24)

*S*(-*z*) → 0, as *z *→ ∞

• Proof of (24):- Applying Brauer's theorem, it can be shown that all the eigen values *λ *of the matrix *E *+ *F *are negative. Hence, *z *= *kλ *> 0.



• Proof of (25):-



From (24) and (25), it is concluded that the numerical method defined by (18a) to (18i) is *L*_0 _stable. Similarly the numerical method defined by (21a) to (21i) is *L*_0 _stable. Since the method is *L*_0 _stable, the discontinuities around *y *= 0 and *y *= *L*_*c *_are not propagated.

## Results and discussion

In order to examine the behavior of the recurrence relations (18a) to (18i) and (21a) to (21i), a series of five numerical experiments, similar to those described in [2], are carried out for two space dimensions. In these experiments the parameter-values used are those used in [2]. For the experiments numbered *n *= 1, 2, 3, 4, and 5, the parameter *C *was given the values 4.1, 19.5, 43.1, 51.4 and 64.0 respectively. The rest of the parameter values are given by

*L*_*s *_= 0.004 *cm*, *L*_*d *_= 0.0320 *cm*, *Lc *= 0.128 *cm*, *L*_*u *_= 0.160 *cm*, *k *= 0.1 *h*, *H *= 0.0004, *κ *= 0.0000018 *cm*^2^/*h*,  = 5.0 *mg*/*ml*, *K*_*D *_= 1.1, *h *= 0.0002, *N *= 19, *Q*_*c *_= 320, *Q*_*u *_= 400, *Q*_*d *_= 80, *b *= 0.5599 *ml*/*mg*.

**Figure 1:**
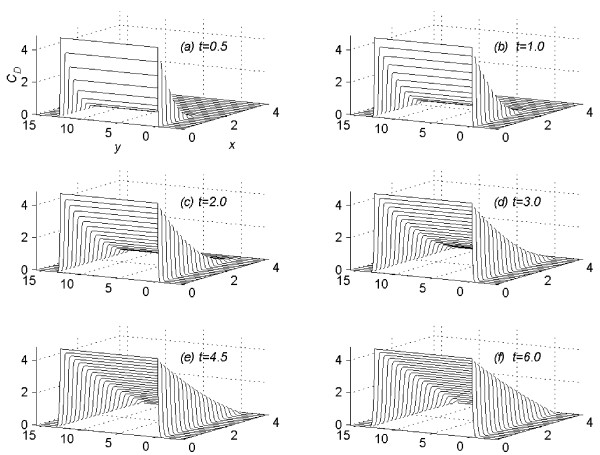
Concentration profiles for *C *= 4.4, viewed as parabolas along mesh lines *x *= x_*l*_, *l *= 0(1)*N*; *t *= 0.5, 1.0, 2.0, 3.0, 4.5, 6.0.

**Figure 2:**
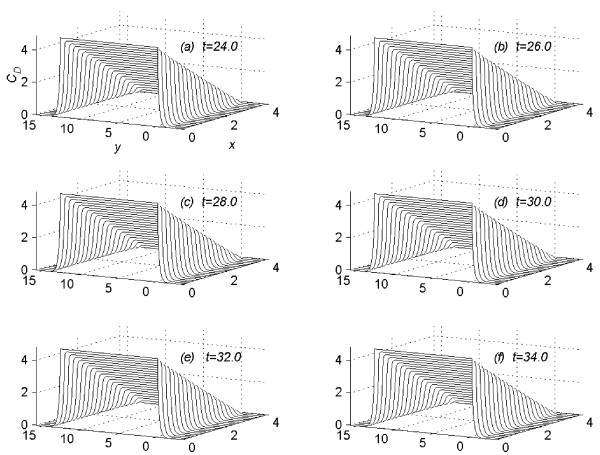
Concentration profiles for *C *= 4.4, viewed as parabolas along mesh lines *x *= x_*l*_, *l *= 0(1)*N*; *t *= 24.0, 26.0, 28.0, 30.0, 32.0, 34.0.

**Figure 3:**
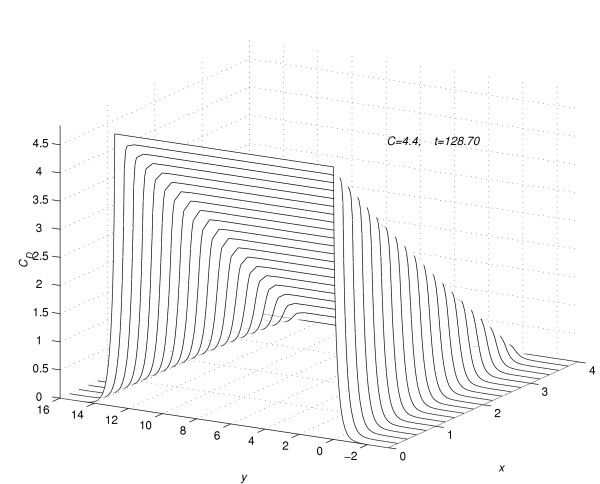
Concentration profiles for *C *= 4.4, viewed as parabolas along mesh lines at *x *= *x*_*l*_, *l *= 0(1)*N*; *t *= 128.7.

It was assumed in all numerical experiments that the drug was applied until steady-state concentration profile is reached. Let  denotes the time in hours, at which the concentration profiles for the *n*^*th *^(*n *= 1, 2, 3, 4 and 5) experiment reaches the steady-state. Suppose that at time *t *= , the vehicle is removed instantaneously from the skin. The pattern of the concentration profile is observed for fifty hours more, after the vehicle is removed at the steady-state. For the *n*^*th *^(*n *= 1(1)5) experiment, the values of *C*_*D *_are computed (1) using the sequential algorithm (18a) to (18i), in the time interval 0 <*t *≤ , (2) using the sequential algorithm (21a) to (21i), in the time interval  <*t *≤  + 50. Following [2], for the value of *n *= 1 (*C *= 4.4), the profiles of concentration *C*_*D *_at *t *= 0.5. 1.0, 2.0, 3.0, 4.5 and 6.0 are given in figure 1. In figure 2, the profiles of concentration *C*_*D *_are given at *t *= 24.0, 26.0, 28.0, 30.0, 32.0 and 34.0. In figure 3 the profile of concentration *C*_*D*_, are given at time *t *=  = 128.7, the time at which concentration, *C*_*D*_, reaches the steady state for the first experiment. In figures 1, 2 and 3, the concentration profiles are viewed as parabolas along mesh lines *x *= x_*l*_, *l *= 0(1) *N*. The figures 1 and 2 show the flow rate in different subregions of the skin region *R*_*s*_, before the concentration reaches the steady state. Figure 3 shows the shape of the concentration profile when the concentration reaches steady-state.

**Figure 4:**
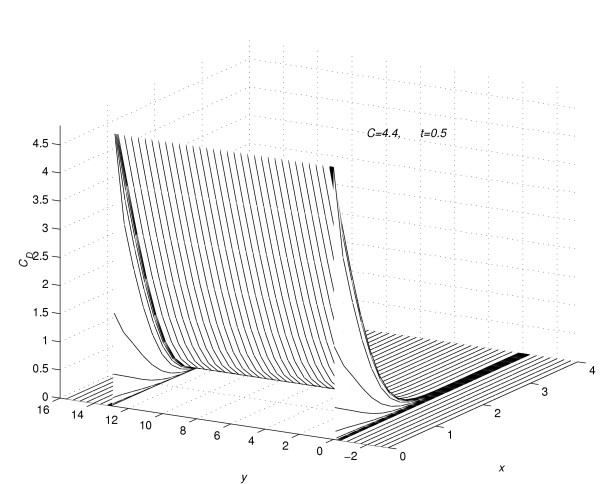
Concentration profiles for *C *= 4.4, viewed as parabolas along mesh lines at *y *= *y*_*m*_, *m *= -*Q*_*d*_(1)*Q*_*u*_; *t *= 0.5.

**Figure 5:**
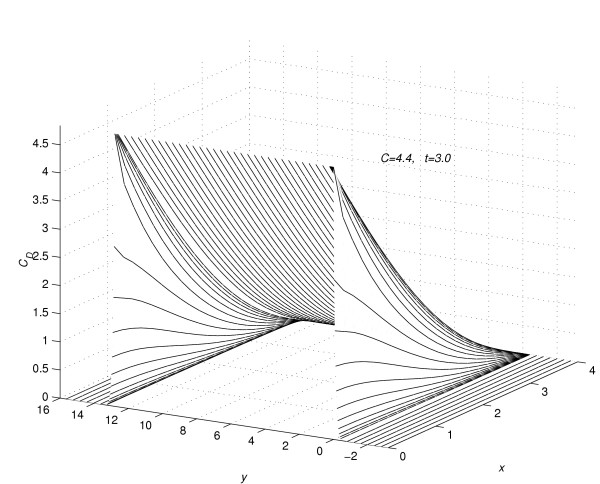
Concentration profiles for *C *= 4.4, viewed as parabolas along mesh lines at *y *= *y*_*m*_, *m *= -*Q*_*d*_(1)*Q*_*u*_; *t *= 3.0.

**Figure 6:**
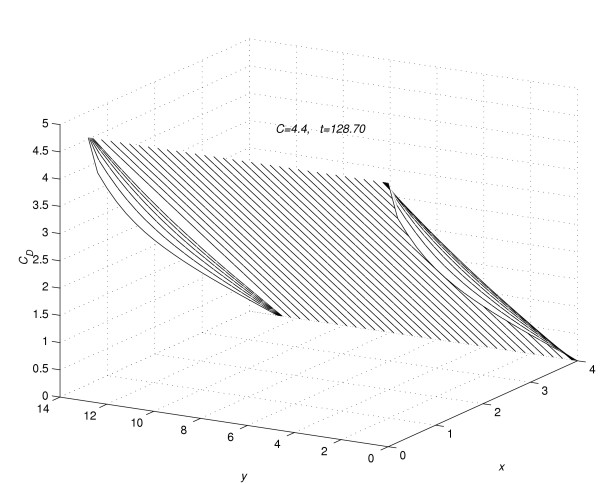
Concentration profiles for *C *= 4.4, viewed as parabolas along mesh lines at *y *= *y*_*m*_, *m *= 0(1)*Q*_*c*_; *t *= 128.7.

In figure 4 and figure 5, for the value of *C *= 4.4, the concentration profiles at *t *= 0.5 and 3.0 are viewed as parabolas along the mesh lines *y *= *y*_*m*_*, m *= -*Q*_*d*_(1)*Q*_*u*_. Figure 4 and figure 5 show the change in shape of the parabolas in the region away from the region of contact of drug and skin. In figure 6, corresponding to the value of *C *= 4.4, the concentration profiles at *t *=  = 128.7 are shown along the mesh lines *y *= *y*_*m*_, *m *= *Q*_*c*_(1)*Q*_*u*_. The figure 6 shows the importance of two-dimensional modelling.

**Figure 7:**
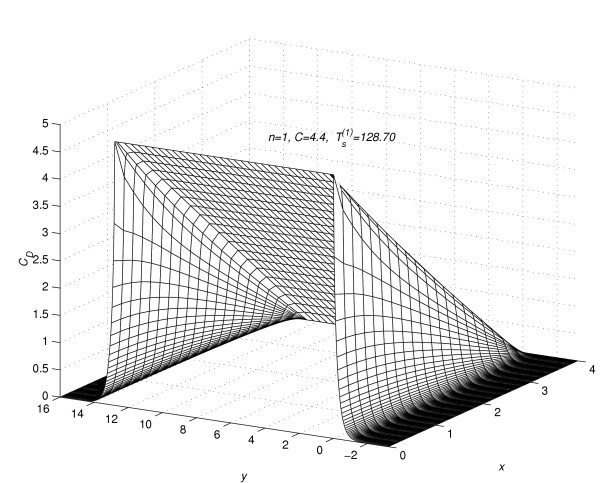
Concentration profiles for *C *= 4.4, when steady-state is reached.

**Figure 8:**
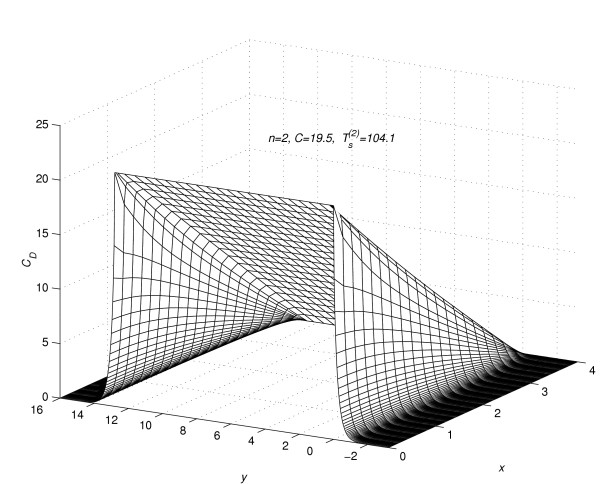
Concentration profiles for *C *= 19.5, when steady-state is reached.

**Figure 9:**
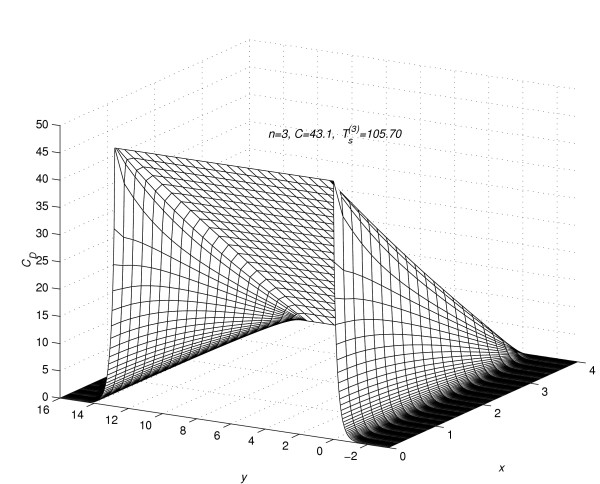
Concentration profiles for *C *= 43.1, when steady-state is reached.

**Figure 10:**
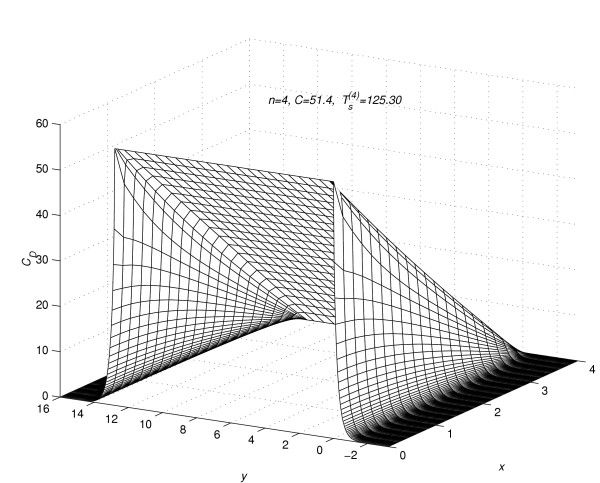
Concentration profiles for *C *= 51.4, when steady-state is reached.

**Figure 11:**
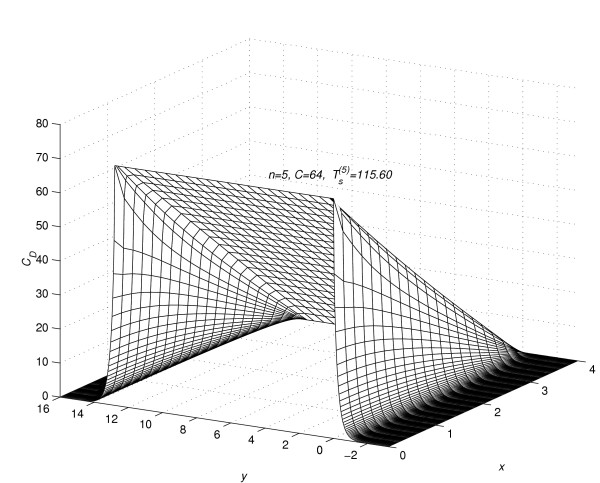
Concentration profiles for *C *= 64.0, when steady-state is reached.

Corresponding to each value of *n *= 1(1)5, the concentration profiles at *t *=  are given in figures 7, 8, 9, 10 and 11, respectively. The above figures show the effect of the value of *C *on the steady-state concentration *C*_*D *_in different regions of *R*_*s*_.

**Figure 12:**
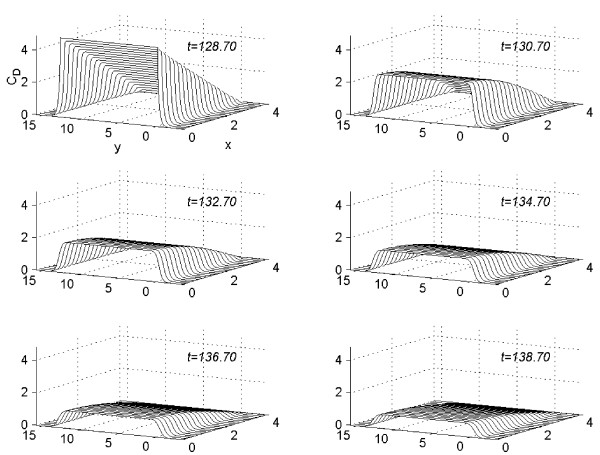
Concentration profiles for *C *= 4.4 viewed as parabolas along mesh lines *x *= x_*l*_, *l *= 0(1)*N*; *t *= 128.7, 130.7, 132.7, 134.7, 136.7, 138.7.

In figure 12, the concentration profiles for the value of *C *= 4.4 are given at *t *= 128.7, 130.7, 132.7, 134.7, 136.7, and 138.7. This graph show the rate of decreasing of the concentration in different regions of *R*_*s *_after the removal of the vehicle.

The *A*_*e *_*versus *time and *J versus *time profiles are monitored for the time interval 0 ≤ *t *≤  + 50 in each experiment.

The values *J *and *A*_*e *_are computed at each time step using the trape-zoidal rule to approximate the integrals in (4) and (5). Thus, to second-order accuracy, for *j *= 1, 2, 3,... etc,



where



and



**Figure 15:**
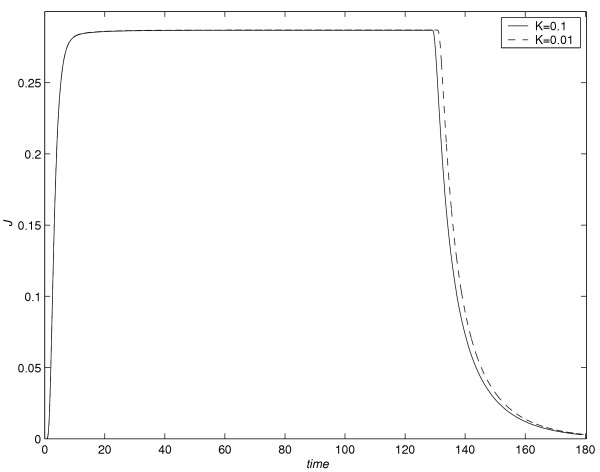
*J versus *time profiles for *C *= 4.4 with *k *= 0.1 and *k *= 0.01.

In literature see [2], the drug absorption experiments are monitored for a large time interval. Hence it is desirable to use a numerical method which gives acceptable results with larger time steps compared to spacial discretization. Since the numerical method used in this section are *L*_0 _stable, it is expected to give accurate results when a large time step is used. Note that the conclusions obtained from all the five experiments are independent of the time step *k*. As an illustration, with *k *= 0.1 and *k *= 0.01, *J versus *time profiles are monitored for the first experiment (*C *= 4.4) and are given in figure 15. The time required to reach the steady-state value of the flux with *k *= 0.1 and *k *= 0.01 respectively are *t *= 128.7 and *t *= 130.37. When the numerical computation is done with *k *= 0.01, the difference in flux during the time interval [128.7 130.37] is 10^-10^, which is negligible. It is also noted that even if the drug is removed at *t *= 130.37 instead of *t *= 128.7, the conclusions drawn in next section remain valid.

## Conclusion

Conclusion is presented in five parts.

### Part 1:- Conclusions based on concentration profiles till steady-state concentration, C_D_, is reached

Concentration profiles at various time levels are examined until steady state is reached. Graphically these concentration profiles are represented as parabolas along mesh lines *x *= *x*_*l*_, *l *= 0(1) *N *or parabolas along mesh lines *y *= *y_m_*, *m *= -*Q*_*d*_(1)*Q*_*u*_. Divide the region of skin, *R*_*s*_, into two mutually exclusive regions *R*_1 _and *R*_2 _as follows.

*R*_1 _= *R*_*c *_× *L*_*s *_and *R*_2 _= *R*_*s *_- *R*_1_.

The following conclusions are drawn based on drug kinetics in the regions *R*_1_, *R*_2 _and *R*_*s*_.

(1) In figure 1 the concentration profiles are viewed as parabolas along mesh lines *x *= *x*_*l*_, *l *= 0(1)*N*. Consider the flow rate of concentration of the drug in the region *R*_1_. From the subplots (a) to (c) of figure 1, it is concluded that during the initial time levels, flow rate towards the skin surface is larger than flow rate towards the skin-capillary boundary. Later-on time levels (from subplots (c) to (f) of figure 1), flow rate towards the skin-capillary boundary becomes larger than flow rate near to the skin surface.

(2) In figure 2 the concentration profiles are viewed as parabolas along mesh

lines *x *= *x*_*l*_, *l *= 0(1)*N*.

From figure 1 and figure 2 it is concluded that in the region *R*_2_, the flow rate near to the skin surface is larger than flow rate towards the skin-capillary boundary.

(3) Consider the flow rate in the whole region *R*_*s*_. From figure 1 and figure 2 it is concluded that, during the initial time levels, flow rate in the region *R*_1 _is larger than the flow rate in the region *R*_2_. But later-on time levels, flow rate in the region *R*_2 _becomes larger than the flow rate in the region *R*_1_, leading to a steady-state concentration profile as given in figure 3. In figure 3 the concentration profiles are viewed as parabolas along mesh lines *x *= *x*_*l*_, *l *= 0(1)*N*.

(4) At any time level *t *= *t*_*j*_, consider the concentration of the drug along the mesh line *x *= *x*_*l*_, *l *= 0(1)*N*. From figure 1, 2 and 3, it is concluded that the concentration of the drug at a mesh point (*x*_*l*_, *y*_*m*_, *t*_*j*_) ∈ *R*_1_, *m *= 0(1)*Q*_*c *_is larger than the concentration of the drug at a mesh point (*x*_*l*_, *y*_*m*_, *t*_*j*_) ∈ *R*_2_, *m *≠ 0(1)*Q*_*c*_.

(5) In figure 4 and figure 5, the concentration profiles are viewed as parabolas along mesh lines *y *= *y*_*m*_, *m *= -*Q*_*d*_(1)*Q*_*u*_. From figure 4 it is concluded that, during the initial time-levels, the shape of the parabolas in the whole region *R*_*s *_retains the same shape as the shape of the concentration profiles given in page 97 of [2]. If a one-dimensional model of drug absorption was sufficient, the following results are expected from [2].

• When steady-state concentration reaches, the shape of the concentration profiles viewed along the mesh lines *y *= *y*_*m*_, *m *= -*Q*_*d*_(1)*Q*_*u *_is same as the shape of the parabolas given in page 97 of [2].

• When steady-state concentration reaches, concentration profiles along the mesh lines *y *= *y*_*m*_, *m *= 0(1)*Q*_*c *_are straight lines.

Contrary to this result, the following results are obtained from two-dimensional modelling.

5.1 From figure 4 and figure 5 it is concluded that, as time increases, the parabolas in the region *R*_2 _have a convex shape, whereas the parabolas given in page 97 of [2] have a concave shape.

5.2 The concentration profiles along the mesh lines, *y *= *y*_*m*_, *m *= 5(1) *Q*_*c *_- 5 are straight lines. These straight lines are shown in figure 6.

5.3 The concentration profiles along the mesh lines, *y *= *y*_*m*_, *m *= 0, 1, 2, 3, 4, *Q*_*c *_- 4, *Q*_*c *_- 3, *Q*_*c *_- 2, *Q*_*c *_- 1 and *Q*_*c *_are not straight lines, but they have the shape as shown in figure 6.

Even though the illustration of figures is done only for one experiment (with *n *= 1, *C *= 4.4), that is only for the first experiment, the conclusions drawn are true for all other experiments (that is, for all values of *C*).

### Part 2:-Conclusions based on concentration profiles when steady-state value of concentration is reached

The steady-state concentration profiles for all the five experiments are given in figure 7, figure 8, figure 9, figure 10 and figure 11. From these steady-state concentration profiles, the following conclusions are drawn.

(1) As the value of *C *increases, the concentration at any point (*x*_*l*_, *y*_*m*_, *t*_*j*_) increases.

(2) As the value of *C *increases, the difference between the concentrations of the drug at mesh points (*x*_*l*_, *y*_*m*_, *t*_*j*_) ∈ *R*_1_, *m *= 0(1)*Q*_*c *_and (x_*l*_, y_*m*_, t_*j*_) ∈ *R*_2_, *m *≠ 0(1)*Qc *increases.

(3) As the value of *C *increases, the difference between the concentrations of the drug at mesh points (*x*_0_, *y*_*m*_, *t*_*j*_) and (*x*_*N*-1_, *y*_*m*_, *t*_*j*_) increases for *m *= -*Q*_*d*_(1)*Q*_*c*_. This increase in difference is more prominent in the region *R*_1 _than in the region *R*_2_.

### Part 3:-Conclusions based on concentration profiles after the removal of the vehicle when steady-state is reached

The vehicle is removed when the concentration reaches steady-state. The concentration profiles are examined for ten more hours, after the vehicle-removal, in an interval of two hours. In figure 12, these concentration profiles are given for *n *= 1 (*C *= 4.4), at time levels *t *=  = 128.7, *t *= 130.7, *t *= 132.7, *t *= 134.7, *t *= 136.7, and 138.7. From figure 12 the following conclusions are made.

(1) After the removal of the vehicle, rate of decrease in the region *R*_1 _is larger than that in *R*_2_.

(2) After the removal of the vehicle, rate of decrease near to the region of skin-surface is larger than that near to region of skin-capillary boundary. Even though the illustration of figures is done only for one experiment (with *n *= 1, *C *= 4.4), that is only for the first experiment, the conclusions drawn are true for all other experiments also.

### Part 4:-Conclusions based on figure 13 (flux *versus* time) and figure 14 (A_e _versus time)

**Figure 13:**
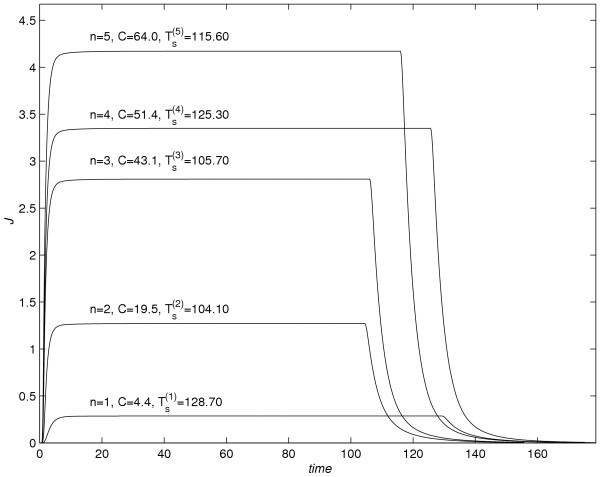
*J versus *time profiles for various values of *C*.

**Figure 14:**
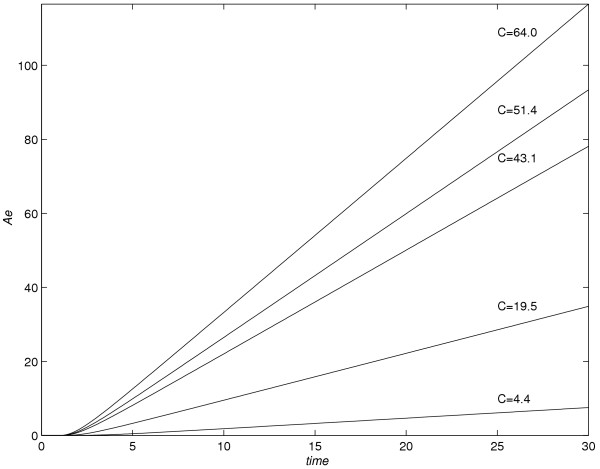
*Ae versus *time profiles for various values of *C*.

The graphs of *J versus *time and *Ae versus *time for the time interval 0 ≤ *t *≤ ( + 50) are shown in figure 13. The following conclusions are drawn based on figure 13.

(1) As the value of *C *increases, the steady-state value of flux increases.

(2) As the value of *C *increases half-life decreases, during the time levels, soon after the vehicle-removal. There is no prolongation of half-life during later-on time, as stated in [2]. It is evident from figure 13 that, the plots of a drug flux *versus *time is a non-linear curve, soon after the vehicle removal. Hence, in experimental studies even when the prolongation of half-life is not seen, the nonlinearity of drug flux *versus *time can be introduced as a characteristic to confirm that dual-sorption model gives an explanation to non-linear kinetic behavior of the drug.

The graphs of *Ae versus *time for the time interval 0 ≤ *t *≤  + 50 are shown in figure 14.

(3) As the value of *C *increases, at any time level *t *= *t*_*j*_, the value of *Ae*(*t*) increases.

(4) As the value of *C *increases, lag-time decreases. The lag-time is computed as t-intercepts of the linear portion of graphs of *Ae versus *time. For various values of *C*, the lag-times are compared with the lag-times given in [2]. Let *T*_*L*1 _denotes the lag-times quoted in [2] as a result of one-dimensional modelling of drug-absorption and *T*_*L*2 _denotes the lag-times obtained as a result of two-dimensional modelling. Table 1 gives the values of *T*_*L*1 _and *T*_*L*2_, corresponding to each value of *C*.

**Table:** 

*C*	4.4	19.5	43.1	51.4	64.0
*T*_*L*1_	3.44	2.29	1.99	1.93	1.84
*T*_*L*2_	3.67	2.52	2.16	2.11	2.04

From Table 1 it is concluded that, for a particular value of *C*, the lag-time obtained as a result of two-dimensional modelling is greater than the lag-time obtained as a result of one-dimensional modelling. For a linear model, lag time is independent of donor concentration *C*. The linear model corresponding to (1) is obtained by substituting  = 0. Its lag time is computed as 1.56 which is greater than 1.44, the lag-time obtained from the one-dimensional linear model (substitute  = 0 in (8) of [2]).

It is an established fact that the drug permeation profiles of a nonlinear model is different from that of a linear model, as a non-linear model assumes that the drug molecules are either dissolved or immobile inside the skin. In this article it is shown that the drug permeation described by the two-dimensional non-linear model is different from one-dimensional non-linear model, as the two-dimensional model takes into account the drug kinetics at the site distant from the area where the ointment is directly applied.
